# Aggressive adenocarcinoma of the lung consisting solely of discohesive cells

**DOI:** 10.1186/1749-8090-8-89

**Published:** 2013-04-15

**Authors:** Yoshiki Kozu, Mitsuhiro Isaka, Yasuhisa Ohde, Takashi Nakajima

**Affiliations:** 1 Division of Thoracic Surgery, Shizuoka Cancer Center, Shizuoka, 411-8777, Japan; 2 Division of Pathology, Shizuoka Cancer Center, Shizuoka, 411-8777, Japan

**Keywords:** Lung adenocarcinoma, Discohesive cells, Pneumonia-like shadow

## Abstract

A 60-year-old Japanese man was found to have diffuse pneumonia-like shadow in the left S^10^ segment on chest computed tomographic scan. Transbronchial lung biopsy yielded a pathological diagnosis of poorly differentiated adenocarcinoma; subsequently, left lower lobectomy was performed. Histopathological analysis showed that the tumor consisted solely of discohesive cells with involvement of the hilar and mediastinal lymph nodes. The immunohistochemical expression of E-cadherin and β-catenin was low, whereas that of p53 was high in the tumor cells. Here, we describe a rare lung adenocarcinoma with discohesive cells, which are considered to indicate high tumor aggressiveness.

## Background

Lung cancers show various radiological presentations, including sporadic pneumonia-like shadow. Lung cancers with this feature are often diagnosed as bronchioloalveolar carcinomas (BACs) or well-differentiated adenocarcinomas with papillary or acinar components. The tumor in the present study, which showed pneumonia-like shadow on computed tomographic (CT) scan, was diagnosed as lung adenocarcinoma consisted solely of discohesive cells that extended diffusely and singly into the alveolar spaces. Herein, we describe this unique lung adenocarcinoma and its associated immunohistochemical findings.

## Case presentation

**Figure 1:**
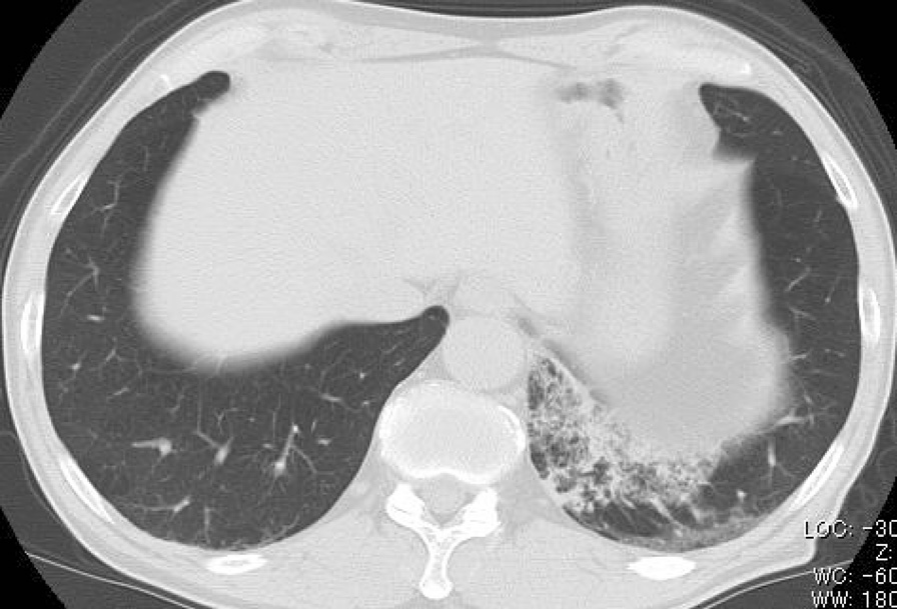
**Chest computed tomographic scan shows diffuse pneumonia-like shadow in the left S**^**10 **^**segment.**

A 60-year-old Japanese man was admitted to the Shizuoka Cancer Center Hospital with the complaints of bloody sputum. For the last 42 years, he had smoked 2 packs of cigarettes a day. He had no history of neoplasm. The preoperative serum carcinoembryonic antigen (CEA) level was elevated to 135.4 ng/mL. No abnormal shadow was seen in the lung field on a chest radiograph. Chest CT scan showed diffuse pneumonia-like shadow in the left S^10^ segment (Figure 1). F18-fluorodeoxyglucose positron emission tomography showed abnormal uptake (standardized uptake value, max 7.1) in the left lower lobe. Sputum cytology showed numerous large atypical cells with large round or oval nuclei, fine chromatin, 1 or 2 prominent nucleoli, and an abundant cytoplasm. Bi-nucleated cells were occasionally observed. These cells rarely formed clusters and were mostly dissociated. Adenocarcinoma was diagnosed based on sputum cytology. Transbronchial lung biopsy yielded a pathological diagnosis of poorly differentiated adenocarcinoma.

**Figure 2:**
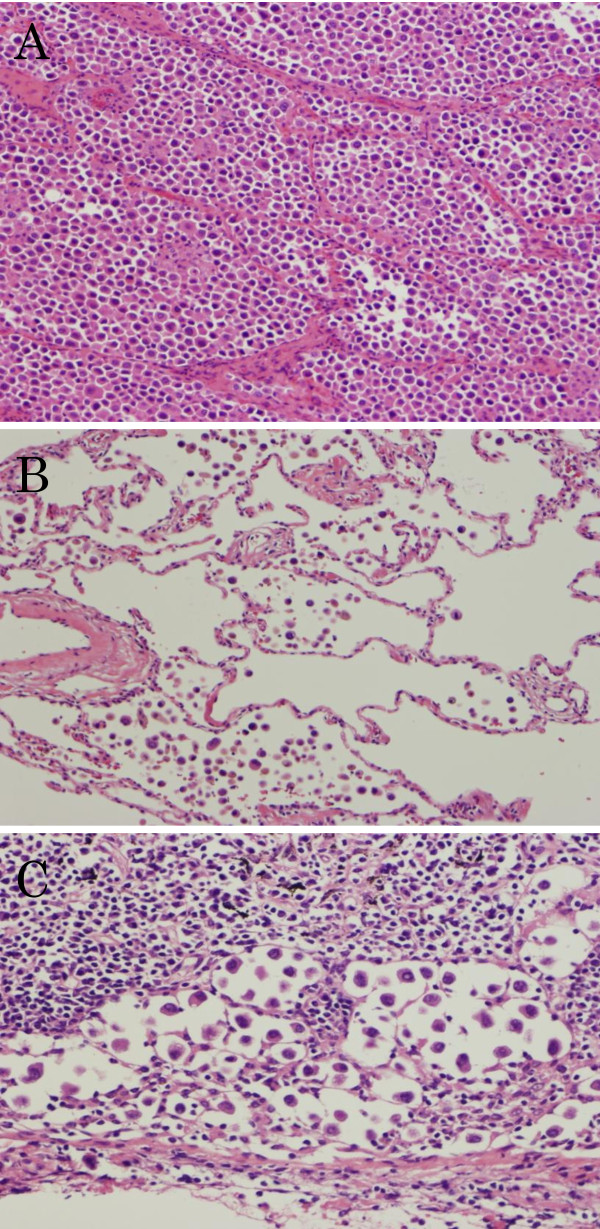
**Histology of the tumor (Hematoxylin and eosin; ×400).** Near the fibrotic focus, the tumor cells pack and fill the alveolar spaces (**A**). Alveolar septae are well preserved. Even in the space where the tumor seems to be absent macroscopically, tumor cells are seen to be diffusely floating in the alveolar spaces (**B**). Dissociated tumor cells invading the peripheral sinus of the lymph nodes singly and diffusely (**C**).

The clinical stage of the adenocarcinoma was T2bN0M0 (stage IIA), and left lower lobectomy was performed. The resected tumor was approximately 6.5 × 4.0 × 3.0 cm in size and occupied most of the S^10^ segment. The cut surface of the tumor was yellowish gray with an indistinct margin and contained a small fibrotic focus in the center. Pleural indentation was not observed. Microscopically, the tumor cells were round and contained hyperchromatic nuclei with a moderate amount of eosinophilic cytoplasm. The tumor cells showed completely discohesive growth and filled the alveolar spaces near the fibrotic focus (Figure 2A). The fibrotic focus was composed of collapsed lung parenchyma without tumor cells. In the tumor outside of the fibrotic focus, tumor cells floated in the alveolar spaces and extended to the entire resected lobe (Figure 2B). The alveolar structure was well preserved. The tumor cells also floated along the small bronchus and diffusely invaded blood vessels and lymphatics. Lymph nodes in the hilar and mediastinal regions were invaded by the tumor. The dissociated tumor cells invaded the peripheral margin of the lymph nodes singly and diffusely, similar to the pattern observed in the peripheral lung (Figure 2C). The pathological stage of the tumor was T3N2M0 (stage IIIA). Alcian blue (AB) - PAS stain produced a weakly positive AB staining on the cell membrane of the tumor cells. Immunohistochemical analysis showed that thyroid transcription factor (TTF)-1 (1:500; DAKO) and p53 (1:100; DAKO) were diffusely positive in tumor cell nuclei. Cytokeratin 7 (1:400; DAKO), napsin A (1:400; IBL) and surfactant apoprotein -A (1:100; DAKO) were diffusely positive, whereas anaplastic lymphoma kinase -1 (1:100; Abcam) was negative in the cell cytoplasm. E-cadherin (1:100; Leica) and β-catenin (1:100; BD Biosciences) were positive on the cell membrane, but their intensity was weaker than that in reactive alveolar cells. Further analysis by electron microscopy (EM) showed that the tumor cells were rich in well-developed microvilli on the cell surface.

Adjuvant therapy was not administered after the surgery because of poor performance status. Six months later, the patient experienced severe headache. His serum CEA level was elevated to 589.9 ng/mL. Brain magnetic resonance imaging showed dilated cerebral ventricles. Lumbar puncture was performed, and cerebrospinal fluid analysis showed meningitis carcinomatosis. Whole-body CT scan showed no evidence of tumor recurrence other than that in the brain. Despite cerebrospinal irradiation, the patient finally died of the disease 11 months after the surgery. Autopsy was not permitted.

## Discussion

The tumor in the present study showed pneumonia-like shadow on CT scan. In lung cancer, this radiological presentation generally leads to the pathological diagnosis of BAC or adenocarcinoma with papillary or acinar components. However, the present case differed completely from other tumor histologies that have been previously described. The tumor consisted solely of discohesive cells without any morphological structure such as a lepidic, papillary, or acinar pattern.

This histological feature is so rare, that few reports describing a similar growth pattern have been published [1-3]. In these reports, discohesive cells were found to be floating diffusely in the alveolar spaces around the central portion harboring papillary components or desmoplastic reaction. Kodama et al. speculated that discohesive cells resemble the hyperplastic cuboidal alveolar cells seen in damaged lungs [1]. Fujita et al. reported that cancer cells were immunopositive for TTF-1, suggesting that these cells were derived from terminal respiratory unit [2]. This immunohistochemical finding was consistent with that observed in our study.

The present case differs from these previously reported cases in 2 respects. First, there were no distinct histological features other than the presence of discohesive cells in the tumor. Even near the central portion, tumor cells showed discohesive growth and filled the alveolar spaces. The second difference was the involvement of the hilar and mediastinal lymph nodes. The pattern of tumor development in the lymph nodes was quite similar to that in the alveolar spaces. Moreover, the development of meningitis carcinomatosis was suggestive of blood-borne metastasis. Therefore, our patient showed 3 routes of cancer metastasis, namely, hematogenous, lymphogenous, and aerogenous metastases.

The tumor aggressiveness observed in the present study can be partly explained by the immunohistochemical findings of low E-cadherin and β-catenin expression and strong p53 expression. Low E-cadherin and β-catenin expression is indicative of reduced intercellular adhesion and strong tumor invasive behavior [4]. Strong p53 expression is also suggestive of tumor invasion and metastasis [5]. Consistent with a previous report [1], we found well-developed microvilli on the tumor cell surface by EM analysis. However, the clinical significance of this microstructure remains unclear.

Lung adenocarcinoma consisting of discohesive cells is not described in the latest WHO classification; therefore, we need to accumulate more data on this subtype and elucidate its clinicopathological features.

## Conclusion

We describe a rare lung adenocarcinoma consisting solely of discohesive cells with its associated immunohistochemical findings. Even after a curative resection, vigilance in surveillance should be maintained because this type of lung adenocarcinoma is considered to indicate high tumor aggressiveness.

## Consent

Written informed consent was obtained from the next of kin of the patient for publication of this case report and any accompanying images. A copy of written consent is available for review by the Editor-in-Chief of this journal.

## Abbreviations

BAC: bronchioloalveolar carcinoma; CT: computed tomographic; CEA: carcinoembryonic antigen; AB: alcian blue; TTF: thyroid transcription factor; EM: electron microscopy; WHO: World Health Organization

## Competing interest

The authors declare that they have no competing interests.

## Authors’ contributions

YK wrote the article and collected data. MI and YO participated in the design of the study and helped to draft the manuscript. TN carried out the histopathological diagnosis. All authors read and approved the final manuscript.
